# Actor feedback and rigorous monitoring: Essential quality assurance tools for testing behavioral interventions with simulation

**DOI:** 10.1371/journal.pone.0233538

**Published:** 2020-05-29

**Authors:** Martha A. Abshire, Xintong Li, Pragyashree Sharma Basyal, Melissa L. Teply, Arun L. Singh, Margaret M. Hayes, Alison E. Turnbull

**Affiliations:** 1 School of Nursing, Johns Hopkins University, Baltimore, Maryland, United States of America; 2 Department of Epidemiology, Bloomberg School of Public Health, Johns Hopkins University, Baltimore, Maryland, United States of America; 3 Outcomes After Critical Illness and Surgery (OACIS) Group, Johns Hopkins University, Baltimore, Maryland, United States of America; 4 Division of Pulmonary and Critical Care Medicine, School of Medicine, Johns Hopkins University, Baltimore, Maryland, United States of America; 5 Division of Geriatrics, Gerontology, and Palliative Medicine, Department of Internal Medicine, University of Nebraska Medical Center, Omaha, Nebraska, United States of America; 6 Division of Pediatric Palliative Medicine, Prisma Health Children’s Hopsital – Upstate, University of South Carolina School of Medicine – Greenville, Greenville, South Carolina, United States of America; 7 Division of Pulmonary, Critical Care and Sleep Medicine, Beth Israel Deaconess Medical Center, Harvard Medical School, Boston, Massachusetts, United States of America; 8 Carl J.Shapiro Institute for Education and Research at Beth Israel Deaconess Medical Center and Harvard Medical School, Boston, Massachusetts, United States of America; University of Alberta, CANADA

## Abstract

**Introduction:**

Simulation is a powerful tool for training and evaluating clinicians. However, few studies have examined the consistency of actor performances during simulation based medical education (SBME). The *Simulated Communication with ICU Proxies* trial (ClinicalTrials.gov NCT02721810) used simulation to evaluate the effect of a behavioral intervention on physician communication. The purpose of this secondary analysis of data generated by the quality assurance team during the trial was to assess how quality assurance monitoring procedures impacted rates of actor errors during simulations.

**Methods:**

The trial used rigorous quality assurance to train actors, evaluate performances, and ensure the intervention was delivered within a standardized environment. The quality assurance team evaluated video recordings and documented errors. Actors received both timely, formative feedback and participated in group feedback sessions.

**Results:**

Error rates varied significantly across three actors (H(2) = 8.22, p = 0.02). In adjusted analyses, there was a decrease in the incidence of actor error over time, and errors decreased sharply after the first group feedback session (Incidence Rate Ratio = 0.25, 95% confidence interval 0.14–0.42).

**Conclusions:**

Rigorous quality assurance procedures may help ensure consistent actor performances during SBME.

## Introduction

Simulation based medical education (SBME), a current mainstay of medical education, provides opportunities for clinical training and evaluation in a safe, controlled environment. It also improves communication, teamwork, and patient outcomes such as time to first compressions in CPR. [1,2] SBME can include low fidelity simple task trainers, high fidelity mannequins, and at the highest fidelity, ‘standardized patients’. [3–5] Typically, standardized patients are actors who are trained to replicate a specific clinical scenario. When simulation is used to test whether an intervention affects clinician behavior in the setting of a randomized trial, consistent actor performances are essential. Because the actors are given the same scenario or script, it is often assumed that their performances are similar. But without excellent consistency, it becomes impossible to determine whether differences in clinician behavior are attributable to the intervention, or to differences in actor performances. In this situation, a rigorous quality assurance process is needed to ensure each actor’s performance adheres closely to the study scenario. While the importance of quality assurance is recognized, few studies have examined the consistency of actor performances over time, and across actors [3,6–8]

The *Simulated Communication with ICU Proxies* (SCIP) study was a double-blind randomized controlled trial (RCT) of an intervention designed to influence ICU physician communication behaviors (ClinicalTrials.gov NCT02721810). [9] The trial tested if ICU physicians (intensivists) randomized to document prognosis for a hypothetical patient at high risk of death were more likely to discuss the option of comfort care in a simulated family meeting. Actors in the trial were required to react and respond to enrolled intensivists according to a detailed script during simulations. Deviations from the script were treated as errors. The aims of this secondary analysis of quality assurance data collected during the SCIP trial was to evaluate the simulation quality assurance program, by 1) comparing the incidence of errors across three actors, 2) determining if the incidence of errors changed over time, and 3) assessing whether the rate of actor errors decreased after group feedback sessions.

## Methods

### Auditions and hiring actors

The SCIP trial hypothesized that physicians who record prognosis for a patient at high risk of death or severely impaired functional recovery prior to a simulated family meeting are more likely to disclose prognosis and offer the option of care focused on comfort during the meeting. The Johns Hopkins Medicine Institutional Review Board approved the study (IRB 00082272). The actors portraying the hypothetical patient’s proxy decision-maker during the simulation, are referred to as Standardized Family Members (SFM). The study team collaborated with the Johns Hopkins Simulation Center to identify experienced, female, African-American SFM between the ages of 50 and 70. During auditions, candidate SFMs participated in an early version of the study scenario. They were also interviewed to discuss availability for rehearsals and study visits, compensation, and previous experiences as a patient or patient proxy. Critical to the auditioning process was the ability of the SFMs to receive critical feedback and incorporate it into their subsequent performances. Selected SFMs were invited to a second audition to test their ability to adapt their performance based on initial feedback. New doctors displaying different behaviors participated in the second round of auditions to demonstrate the diversity of physician behaviors the actors might encounter during the trial. Finally, three SFMs were hired based on performances, responsiveness to feedback, and scheduling availability. All hired SFMs lived in the Baltimore metro-region and had personal experiences advocating for hospitalized family members. Three SFMs were hired to ensure availability at a convenient time for study participants including evenings and weekends which was critical to meeting the trial’s recruitment target. However, hiring multiple SFMs created a need for vigilance regarding standardization of the performance and fidelity to the study script.

### Developing the patient scenario

The scenario described an 81-year-old, African-American male with acute respiratory distress syndrome (ARDS) complicated by septic shock on day 3 of treatment in a medical ICU. The patient’s probability of in-patient mortality at admission was estimated as 64% using APACHE III. [10] By ICU day 3 his probability of in-patient mortality had climbed to 88% according to the Mortality Probability Model II-72 hours. [11,12] Although these model-based estimates were not provided to study participants, the scenario provided clear indicators that the patient was at high risk of in-hospital mortality and likely to require 24-hour nursing care in a residential facility if he survived hospitalization. The scenario was presented to physicians in a paper chart that included flow sheets, physical examination findings, past medical history, laboratory values, ventilator settings, radiology report, and summary assessment and plans from the first three days of ICU admission. All documents and data were de-identified and adapted from the medical record of an actual patient to enhance realism of the scenario.

### Developing the simulation script

A high fidelity simulation was essential to ensure valid trial results. The intervention being tested was expected to have the greatest effect on physician behavior during meetings with passive family members with low health literacy. Therefore, the simulation script called for the SFM to portray a daughter who had minimal understanding of her father’s clinical condition and was unaware that her father was sick enough to die at the meeting’s outset. The script called for the daughter to be passive, deferential, and to neither question nor challenge information provided by physicians. If the physician disclosed that the patient was sick enough to die, the scripted response was surprise and emotion, followed by a *single* clarifying question: “What do you think is most likely to happen?”

Study investigators created a list of 20 questions and statements that enrolled ICU physicians were likely to exhibit based on previous studies of ICU physician behavior, [13–15] and clinical experience. Responses to each statement and question were then drafted based on published research, [16,17] and clinical experience. To further ensure a high degree of realism, quotes from transcripts of actual ICU family meetings recorded during a prior study [18], were included in the script as examples of authentic proxy responses. The script also included background information on the psychosocial and health history of the hypothetical patient and his daughter (see Table 1, S1 and S2 Tables). Finally, the script was reviewed by the SFMs who edited responses for authenticity to the local Baltimore colloquialisms/vernacular.

**Table 1:** Association between additional simulation performances and error incidence rate.

	Total errors	P-value	Major errors	P-value
**Crude IRR**	0.97 (0.94–0.99)	0.003	0.97 (0.95–1.00)	0.08
Adjusted IRR*	0.95 (0.93–0.97)	<0.001	0.96 (0.93–0.98)	<0.001

IRR: Incidence rate ratio

*Model adjusted for SFM and whether the physician disclosed that the patient was sick enough to die.

### Actor training

The first step in actors’ training was a read-through and discussion of the script with the study team. Second, each actor participated in a video-recorded rehearsal simulation with a physician who was ineligible for the study. Third, the study team reviewed the rehearsal videos with the actors as a group and provided feedback to help the actors identify physician statements requiring a scripted response. Steps 2 and 3 (rehearsal simulation and feedback session) were repeated 3 times until the study team was confident that actors could portray realistic emotional responses, adhere to the script, and provide appropriate scripted responses to key physician statements.

### Roles and responsibilities of the quality assurance team

Two clinical fellows were recruited to serve as a Quality Assurance (QA) team tasked with monitoring actor performances during study simulations. In addition to helping draft the script, the QA team participated in SFM training, reviewed each simulation to identify actor errors, and decided if each error could have influenced the enrolled physician’s behavior during the simulation. Errors which the QA team believed had the potential to influence one of the main study outcomes were designated as “major” errors. For example, when a SFM introduced herself and said “I guess I’m the decision-maker” this was marked as a minor error and the SFM was instructed to reply “I’m his only child” instead. While referring to oneself as a decision-maker was unlikely to change how the physician behaved, the study script called for the SFM to portray a family member who is unaware of how proxy decision makers are identified and unfamiliar with medical jargon. In contrast, the SFM response “Umm…I don’t know what all that means,” was marked as a major error since the study script instructed SFMs not to seek clarification, and doing so could have resulted in the physician providing clear information about prognosis which was a main study outcome. An operations manual for reviewing simulation videos was developed by the QA team to ensure consistency over the year-long course of the study.

Both members of the quality assurance team independently reviewed the video recordings as well as the written transcription of each simulated family meeting. They used a Research Electronic Data Capture (REDCap) form to record performance evaluations via a standardized questionnaire. [19] Then, the Data Comparison Tool for Double Data Entry in REDCap was used to compare responses and identify discrepancies. Discrepancies were resolved via discussion between the QA Team, Principal Investigator, and Research Study Coordinator.

### Performance feedback

**Fig 1:**
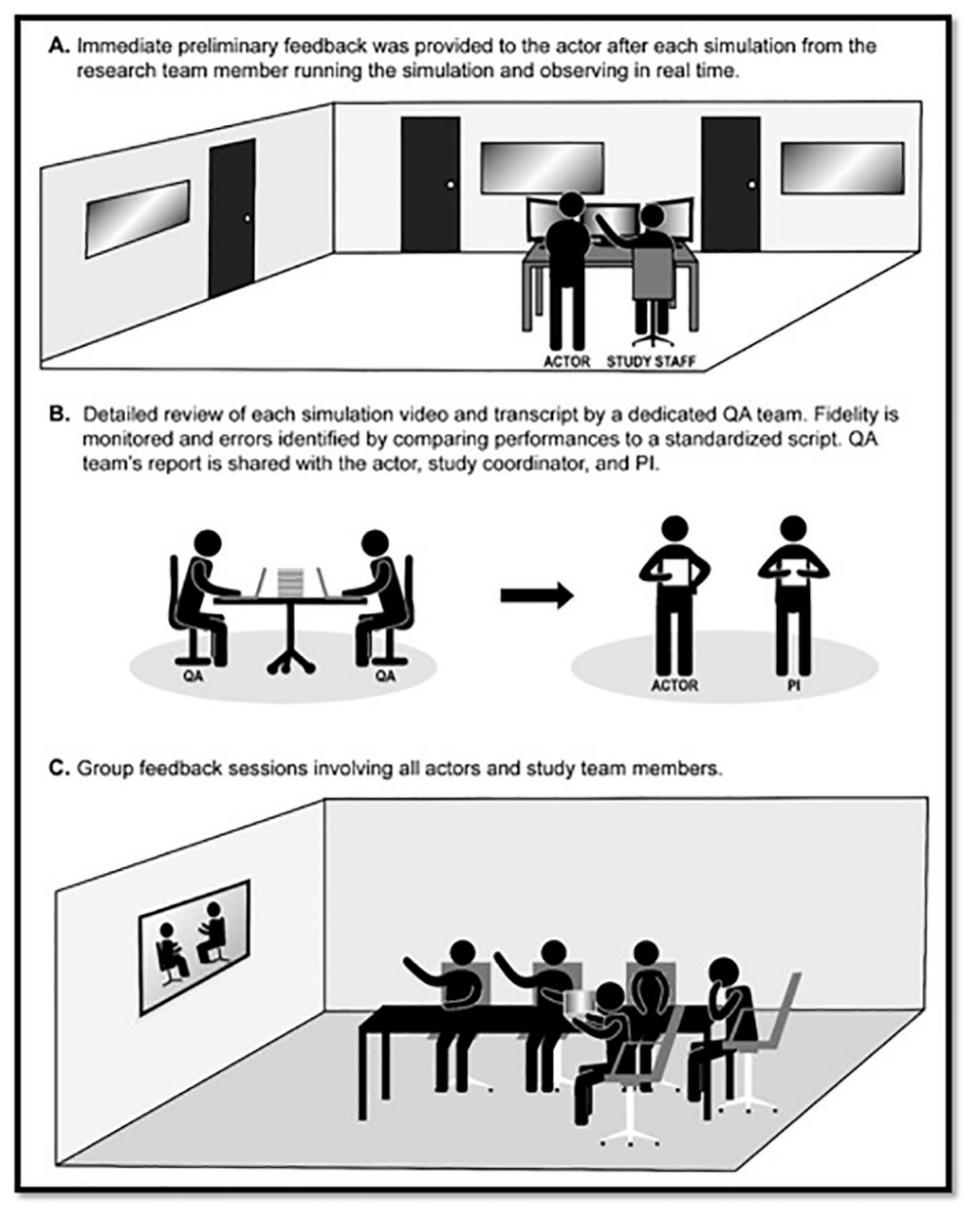
The three modes of feedback used to standardized actor (SFM) performances. Abbreviations: PI, Principal Investigator; QA, Quality Assurance; SFM, Standardized Family Members.

Actors received feedback on their performances throughout the trial in three ways (Fig 1). First, each simulation was run by a study team member (principal investigator, study coordinator, or research assistant) who provided immediate feedback to the actor based on real-time observation of the simulation.

Second, the written report generated by the QA team was shared with the actor, study coordinator, and principal investigator. Each written report highlighted exactly where in the simulation transcript any errors occurred, and suggested how the actor ideally should have responded (see Table 2,S1 and S2 Tables). After receiving these reports, actors had the opportunity to review the video and transcripts of their performances and discuss the report with the Principal Investigator or Research Study Coordinator. To minimize the risk of bias and preserve anonymity, QA team members did not communicate directly with SFMs. Finally, three 60-minute group feedback sessions involving all actors, the Principal Investigator, and the Research Study Coordinator were scheduled. Sessions were conducted after 9 simulations (group feedback 1), 32 simulations cumulatively (group feedback 2) and 77 cumulative simulations (group feedback 3). The first group feedback session was scheduled to ensure each actor had completed at least 2 study simulations prior to meeting.

**Table 2:** Association between study periods and error incidence rate.

	Period 0	Period 1	Period 2	Period 3	P for trend
**Total errors**					
Crude IRR	Ref	0.37 (0.22–0.61)	0.20 (0.11–0.36)	0.19 (0.10–0.36)	<0.001
Adjusted IRR*	Ref	0.39 (0.23–0.66)	0.19 (0.12–0.32)	0.16 (0.09–0.27)	<0.001
**Major errors**					
Crude IRR	Ref	0.21 (0.09–0.50)	0.22 (0.11–0.45)	0.17 (0.08–0.37)	<0.001
Adjusted IRR*	Ref	0.25 (0.14–0.42)	0.21 (0.13–0.35)	0.12 (0.07–0.13)	<0.001

IRR: incidence rate ratio

* Model adjusted for SFM and whether the physician disclosed that the patient was sick enough to die.

Group feedback sessions provided a forum for actors to discuss improvised responses to unexpected questions not addressed in the study script and offer each other suggestions for handling challenging situations. In addition, feedback sessions were used to review summarized feedback provided by the participating doctors on perceived conflict and realism of the simulation, to update the actors regarding additions or amendments to the study script, and to point out trends or concerns identified by the QA team. These group sessions also served as an opportunity for actors to share their experiences with one another and discuss techniques they had developed for recognizing intensivist behaviors requiring specific responses.

### Statistical analyses

**Fig 2:**
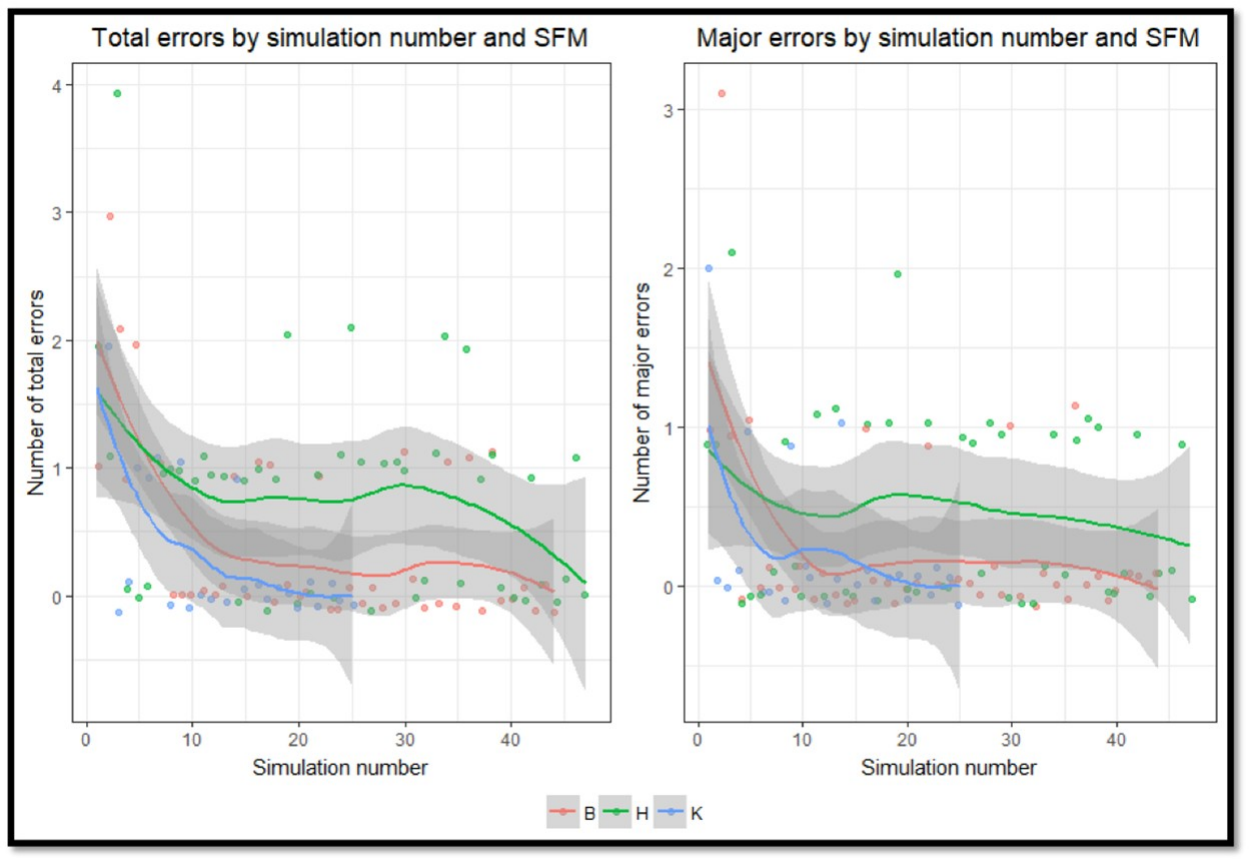
Number of errors in sequential simulations performed by each of the three standardized family members (SFM). The SFM are represented by their first initials B, H, and K. Points are jittered to improve visualization. Trend for each SFM is shown using loess smoothing with 95% confidence intervals.

**Fig 3:**
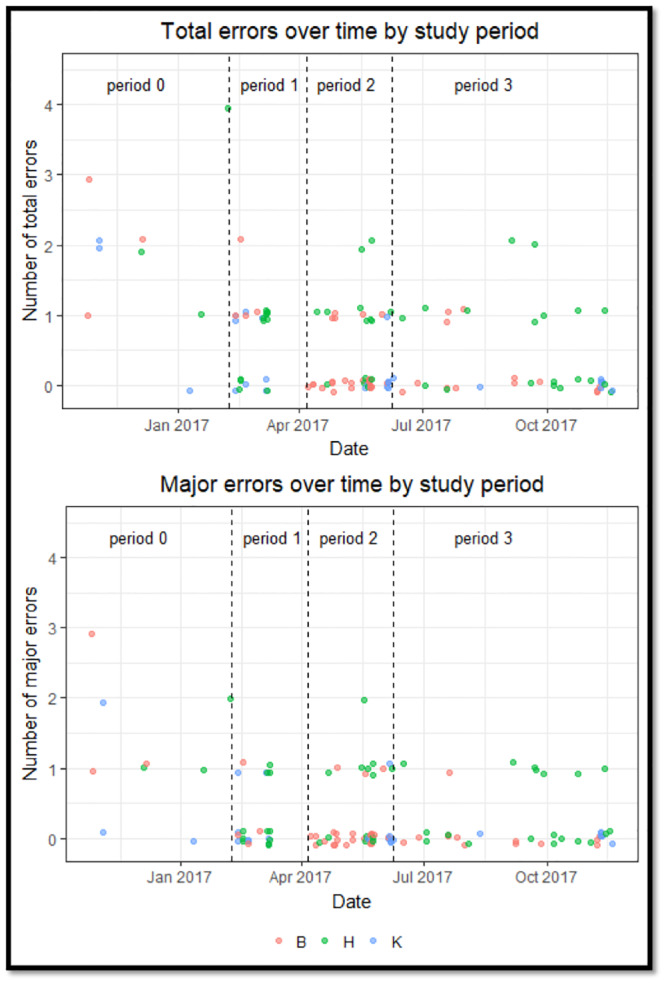
Number of errors in each simulation by calendar date. Point colors correspond to the three standardized family members who are represented by their first initials B, H, and K.

The primary outcome of interest was the number of SFM errors identified by the QA team in each simulation. All analyses were repeated using the subset of errors identified by the QA team as having potential to influence an enrolled physician’s behavior during the simulation. This subset of errors were designated as “major errors.” Error rates across the three SFMs were visualized using violin plots, and then compared using the Kruskal-Wallis rank sum test. For exploratory purposes, the incidence of errors over time was displayed by plotting the number of errors in each simulation sequentially for each SFM (Fig 2), and then versus calendar date (Fig 3). Fig 2 includes loess-smoothed plots of each SFM’s performance errors, and 95% confidence intervals calculated using bootstrapped standard errors.

To assess whether the risk of a SFM committing errors decreased with practice, we regressed total errors on the number of simulations each SFM had performed. To assess whether group feedback sessions decreased the incidence rate of errors, errors were regressed on an variable indicating the number of group feedback sessions which had occurred. Calendar dates were grouped into four periods corresponding to dates before any group feedback sessions (Period 0), dates between the first and second group feedback session (Period 1), etc. Dates before the first group feedback session were used as the reference period.

Poisson regression was used to estimate incidence rate ratios [20, 21] with standard errors computed via the Delta method. Models were adjusted for the SFM performing in the simulation and whether the physician disclosed that the patient was sick enough to die. Physician disclosure that the patient was sick enough to die was included in adjusted models because SFMs were required to perform an emotional response in response to disclosure and ask the scripted clarifying question which increased the likelihood of error. The null hypothesis of equidispersion (mean equal to variance) was tested for each model. Analyses were repeated using major errors as the dependent variable as a sensitivity analysis. All analyses were performed with R 3.6.0 (R Core Team (2019). Vienna, Austria).

## Results

The three SFMs performed in 44, 47, and 25 simulations respectively due to physician scheduling and differences in SFM availability. There was a statistically significant difference between the total error rates of the three SFMs (H(2) = 8.22, p = 0.02), and the rates of major errors committed (H(2) = 8.33, p = 0.02) (see Fig 1, S1 and S2 Tables). The number of errors in each sequential simulation stratified by SFM is displayed in Fig 2 with the greatest decline apparent during the first 12 simulations performed by each SFM. The timing of group feedback sessions and errors during simulations is presented in Fig 3 and shows that frequency of errors decreased after the first group feedback session and then remained relatively constant.

In adjusted analyses, each additional performance by a SFM was associated with a statistically significant decrease in the risk of committing an error (Incidence Rate Ratio[IRR] = 0.95, 95% confidence interval [CI] 0.93–0.97, P<0.001) and in the risk of commiting a major error (IRR = 0.96, 95% CI 0.93–0.98, P<0.001) (Table 1). The incidence of errors in simulations performed after one, two, or three group feedback sessions (periods 1–3) were also significantly lower than the incidence prior to the first group feedback session (Table 2). This improvement was most evident after the first group feedback session when the adjusted incidence rate of major errors decreased by 75% (IRR = 0.25, 95% CI = 0.14–0.42).

## Discussion

SBME is a well-established tool used for formative assessment of healthcare providers and is gaining popularity as a summative assessment tool in some settings. [22] SBME improves development of technical procedural skills, critical thinking, and communication strategies without threat of patient harm. [8,22] However, consistent actor performances are critical in trials of behavioral interventions. In this secondary analysis of the SCIP trial we have presented a detailed QA protocol for assuring consistent SFM performances in the setting of a randomized trial. Error rates varied between SFMs, but improved with repitition, and improved significantly after the first group feedback session.

Without consistent, high-fidelity actor performances, simulation may be less effective for both teaching and testing interventions. In the setting of a randomized trial, it is not possible to know during the design phase what magnitude of effect an intervention will have, or how errors and inconsistencies in a simulation will affect the outcome. For an intervention with a small effect size, the bias introduced by variability in actor performances may be sufficient to change how the trial results are interpreted. Therefore, we viewed developing a rigorous QA program as a worthwhile opportunity to reduce variability in the study environment. In this way, reducing errors is similar to calibrating laboratory equipment.

While significant effort has gone toward fostering consistency in the way learners are equaluated, comparatively little research has been conducted into best practices for standardizing SBME performances. [6–8,23,24] In the most recent guidelines for reporting on SBME simulation, no standards were provided for describing the simulation aside from theoretical, conceptual, and situation-specific exposures. The guidelines also did not include recommendations for methods to address intervention fidelity, nor to address reproducibility, training, and assessment of actor performances. [23] The protocol outlined above provides an example framework for QA design and reporting, although further contributions to methodologic considerations for simulation are needed.

Simulation programs often struggle with maintaining a highly qualified and thoroughly trained workforce. [3] This is attributable to the intermittant nature of work, variations in institutional support of programs, and the high demand for simulation experiences as part of educational curricula and hospital-based learning. Many programs have informal monitoring methods, but financial constraints often limit the time and resources available for QA and feedback. Our findings suggest that rigorous QA is a useful tool for standardizing simulations and this may be particularly important when actors are assigned to perform in multiple scenarios, or have minimal experience. We recognize many programs will not be able to support a dedicated, 2-person QA team in addition to immediate feedback from the study or teaching team. Therefore we suggest designing a feasible QA program tailored to each program’s specific sitaution. Based on our experience, we suggest that the minimal components for a feasible QA program include a group feedback session after most actors have performed in at least 10 simulations and individualized feedback on a random sample of 10% of all simulation performances evaluated by a dedicated reviewer throughout a study or project.

Although our data did not allow us to estimate the impact of immediate feedback on error rates, we did demonstrate that a group feedback session was effective to reduce actor error. In our study, we offered this first session after each actor completed a rigorous training and a small number of simulations. The relatively high observed error rate prior to this first group feedback session suggest that rehearsal simulations did not completely prepare the study actors for some of the situations encountered within the trial. Based on our experiences, we believe providing feedback to actors is similar to providing feedback to colleagues in the clinical environment or participants in a simulation. [25] We propose strategies to maximize the effectiveness of a simulation QA program in Box 1.

**Box 1. Strategies to maximize the effectiveness of a simulation QA program.**

- Set clear expectations about the nature and frequency of feedback
- Critique the performance, not the person
- Be self-aware while providing feedback–manage tone, body language, biases etc.
- Use pronouns that are inclusive (e.g. We are working together)
- Ask open-ended questions to understand barriers to performance
- Establish consistency and credibility in the observation approach
- Provide actors with an approved way to challenge feedback when they disagree
- Always explain what approach or action is preferred, not just what went wrong

This study has several limitations. Although, the QA methodology for this study was designed a priori, this is a secondary analysis of data from a randomized trial that was not designed specifically to evaluate the causal effect of any specific component of our QA program. There were also only 3 actors involved in the study, and simulations were assigned to actors based on availability and scheduling. Finally, we used separate models to describe changes in error rates over time and in relation to feedback sessions, and did not attempt to simultaneously model the impacts of these two exposures. The study’s strengths include a rigorous quantitative evaluation of a QA plan deployed over more than 100 simulations in the course of a year in the setting of a randomized trial where consistency was essential. In conclusion, we have provided a methodology for QA in SBME and trials utilizing simulation and demonstrated that such methods can reduce errors in actor performances in a small sample. Future studies should continue to employ and test QA methods to strengthen the value of simulation in healthcare.

## Supporting information

S1 Table Background information on the hypothetical patient and proxy provided to actors. (DOCX)

S2 Table Example of written QA review including dialogue, errors and feedback. (DOCX)

S1 Fig Distribution of total errors and major errors by standardized family member. (TIF)
