# Supplementary material for: Actor feedback and rigorous monitoring: Essential quality assurance tools for testing behavioral interventions with simulation
Source: PLoS One. 2020 May 29;15(5):e0233538. doi: 10.1371/journal.pone.0233538 (PMC7259593; doi:10.1371/journal.pone.0233538)
Supplement: S1 Table — (DOCX) [file pone.0233538.s001.docx]

| **SDM Table 1: Background Information on the Hypothetical Patient and Proxy Provided to Actors** | |
| --- | --- |
| **Clyde D. Samuels: Patient** | |
| **Age** | 81 (dob: 6/13/1934) |
| **Residence** | Northwood neighborhood, Baltimore City, lives alone with help from his daughter Dorothy |
| **Marital Status** | Divorced since 1975 - no contact with wife in decades |
| **Spirituality** | Christmas and Easter services mainly. Doesn't talk about it. |
| **Employment** | Machineist at the Breeze Point plant of Western Electric Corp (parts manufacturing) until plant closure in 1983. Occasional part-time work until 1990. |
| **ADLs** | Able to bath, dress, transfer, toilet, and feed himself. All ADLs intact. |
| **IADLs** | Uses the microwave and toaster to re-heat food, uses the telephone (land line), and still occasionally drives himself places. Manages his own medications. Dorothy (his daughter) does his laundry, helps with grocery shopping and housekeeping, cooks meals for him frequently, schedules most of his appointments, and keeps tabs on his finances via on-line banking. |
| **Leisure** | Watches a lot of TV. Occasionally visits old friends. Likes going out for seafood in the summer. Has an extensive collection of cassette tapes. Took the bus for a weekend in Ocean City each summer until about 3 years ago. |
| **Dorothy Weems: Daughter** | |
| **Age** | 60 (dob: 10/02/1955) |
| **Residence** | Waverly, Baltimore City - 20 minute walk and 5 minute drive from Clyde |
| **Marital Status** | Married |
| **Family** | Lives with husband, an adult daughter, and 1 school-aged grandchild. Has a son in the military who lives on-base in California with wife and 3 kids. Youngest son is in North Carolina and hasn't been home in many years for reasons Dorothy does not discuss. |
| **Spirituality** | Irregular Sunday services at Southern Baptist Church (Clifton neighborhood - Reverend Hickman) |
| **Employment** | Customer Service Center at Baltimore Gas & Electric (BG&E). Counting down the months till she can retire. |
| **Education** | Graduate of Eastern High School (BCPS) |
| **Relationship with Clyde** | Helps care for her father (Clyde) out of both love and duty. Remembers him as a fairly "hands-off" father who supported his family financially but left day-to-day household management to his wife. Clyde is generally good natured and supportive of his daughter's decisions, but he can be stubborn and clings fiercely to his independence. Dorothy's been trying to convince him to quit driving for years but Clyde refuses to give up his keys. She wishes he'd get rid of the dusty cassette tapes filling the guest room. |
| **Understanding of Clyde's medical history** | Dorothy knows Clyde can't have certain foods and needs to keep track of his meds because he has diabetes. She understands his new kidney didn't work right. He gets a little less steady on his feet and a little more forgetful each year but Dorothy doesn't consider Clyde to be sick. |
| **Previous experiences with critical illness and disability** | Dorothy helped care for her father after his kidney transplant about a decade ago. The transplant went smoothly but Clyde was not an easy patient to nurse.   Dorothy's father-in-law died of Parkinson’s in 1998. Although her sister-in-law managed his care including placement in a long-term care facility during his last year of life, Dorothy witnessed her father-in-law's deterioration and the difficulty of finding acceptable and affordable nursing care. |
