# Supplementary material for: Actor feedback and rigorous monitoring: Essential quality assurance tools for testing behavioral interventions with simulation
Source: PLoS One. 2020 May 29;15(5):e0233538. doi: 10.1371/journal.pone.0233538 (PMC7259593; doi:10.1371/journal.pone.0233538)
Supplement: S2 Table — (DOCX) [file pone.0233538.s002.docx]

**SDM Table 2: Example of Written QA Review including Dialogue, Errors and Feedback**

| - **Doctor’s vague statement about prognosis**: None - **Doctor’s explicit statement about prognosis**: Yes Time: 7:30   Line(s): 134-135 MD: “He’s now had failure of his lungs, he’s had failure of his cardiovascular system, so we’re supporting both of those with types of life support and now he’s moving towards failure of the kidney system, so a third organ system failure, which puts him at a very high risk for both death and a prolonged stay in the ICU.”   - **Doctor clearly explains that Clyde is unlikely to regain independence if he survives**: Yes Time: 8:00   Line(s): 143 MD: “We can never put exact numbers on it, but in a case like his with his age and his immune status from the kidney, the chance of him leaving the hospital and going back to a state where he was before, living independently, that is very, very low.” |
| --- |
| **Feedback to SFM: 1. Major Script deviation:** In line 127,  **MD:** “So, some people would say let’s keep. . . I want you to keep going with all the machines and all the medicines necessary whereas some other people would say, no. I actually don’t want those things and when it’s my time it’s my time. So, did you ever have a discussion with him about that? “  **Dorothy:** I know he wouldn’t want to be on machines.  **Correction:** Reply, "No" in response to the question "Did you have a discussion like that?"  **2. Minor Script deviation:** Multiple times throughout the session, Dorothy refers to talking to family. In line 187,  Dorothy says, “I guess I should call my sons and my daughter.”  **Correction:** Do not bring up talking to family members unless the MD has clearly explained possible treatment options and asked Dorothy to make a decision. |
