# Supplementary figures and images for: Actor feedback and rigorous monitoring: Essential quality assurance tools for testing behavioral interventions with simulation
Source: PLoS One. 2020 May 29;15(5):e0233538. doi: 10.1371/journal.pone.0233538 (PMC7259593; doi:10.1371/journal.pone.0233538)

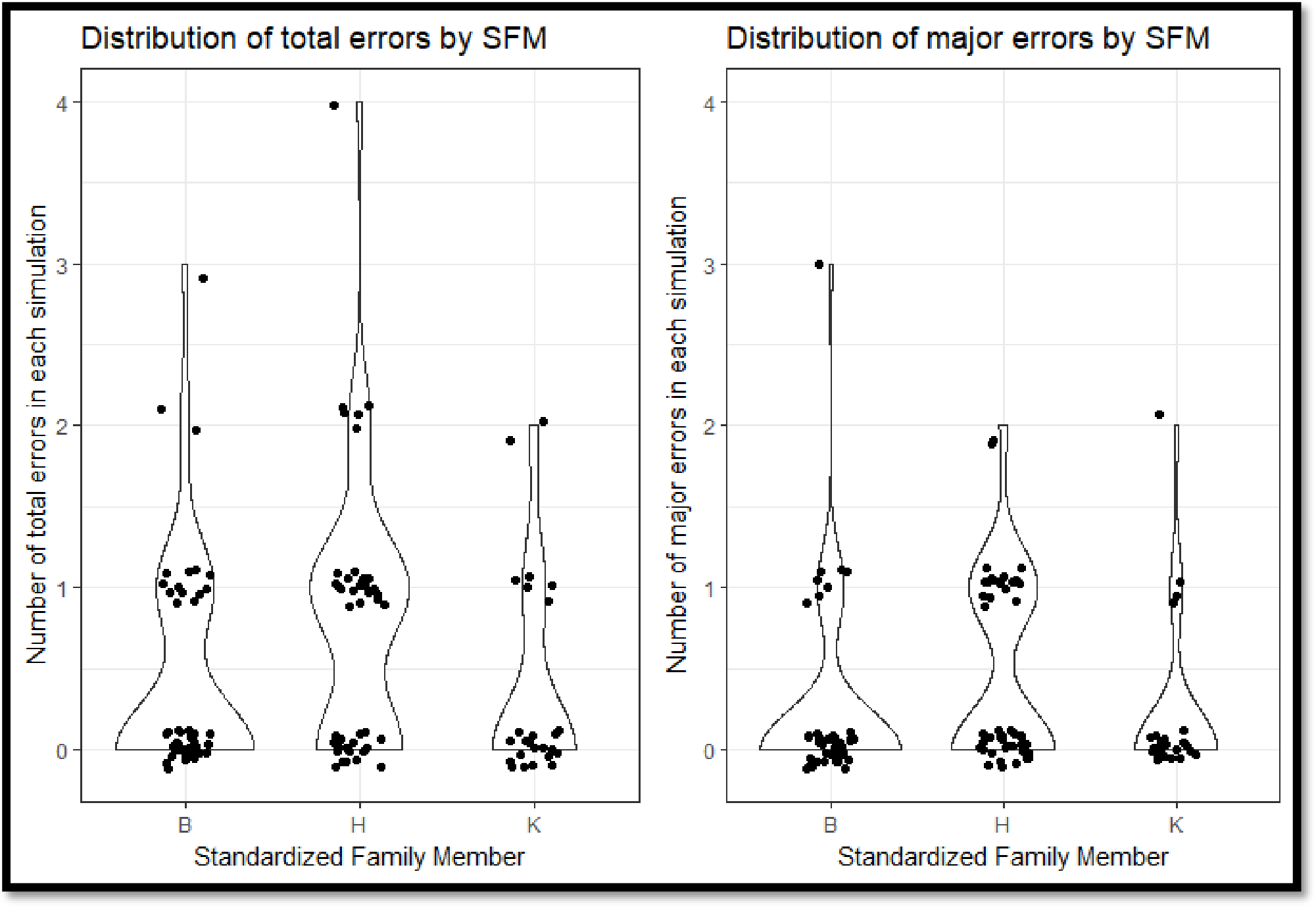

Supplement: S1 Fig — (TIF) [file pone.0233538.s003.tif]
